# Surgery of diaphragm paralysis in patient with severe dyspnea after coronary artery bypass grafting with aortic valve replacement

**DOI:** 10.1186/1749-8090-10-S1-A52

**Published:** 2015-12-16

**Authors:** Dmitrii Tungusov, Igor Chernov, Dmitrii Tarasov, Dmitrii Kondratyev, Vadim Pasyuga, Anna Motreva, Sergey Ekimov, Maxim Isaev

**Affiliations:** 1 Federal Centre for Cardiovascular Surgery, Astrakhan, 414024, Russia

## Background/Introduction

Unilateral diaphragm paralysis is an often not recognized cause of dyspnea especially after cardiac surgery procedures. In some cases diaphragm paralysis disables to wean patient from ventilation. Surgical treatment of unilateral diaphragm paralysis has been described in case reports and in small series since 1985.

## Aims/Objectives

We present a 56-years-old female with left phrenic nerve paralysis after coronary artery bypass grafting with aortic valve replacement.

## Method

The patient was extubated in 6 hours after the operation. During next two days progressive dyspnea was observed and patient became ventilation dependent. Six attempts to wean patient from ventilator failed. Elevation of diaphragm up to the 3rd rib have been registered. Since there was no improvement after respiratory muscle training, plication of the hemidiaphragm was performed by a left thoracotomy.

## Results

The patient improved with regard to respiratory complaints and lung function. Extubation have been performed in 12 hours after diaphragm plication. In 3 days patient was transferred from ICU and later on discharged from the hospital free from dyspnea, angina and heart insufficiency. Their quality of life is greatly improved. The patient is now in follow up for 4 months.

## Discussion/Conclusion

The success of surgical treatment depends on preoperative selection of patients. It is necessary to exclude all other reasons responsible to dyspnea. Although comparison of the available data of diaphragm plication seems an effective and safe procedure for patients with symptomatic, acquired unilateral diaphragm paralysis.

## Consent

Written informed consent was obtained from the patient for publication of this abstract and any accompanying images. A copy of the written consent is available for review by the Editor of this journal.

